# Clinicopathological and Prognostic Significance of the EML4-ALK Translocation and IGFR1, TTF1, Napsin A Expression in Patients with Lung Adenocarcinoma

**DOI:** 10.5146/tjpath.2020.01503

**Published:** 2021-01-15

**Authors:** Pınar Bulutay, Nalan Akyürek, Leyla Memış

**Affiliations:** Department of Pathology, Koç University Hospital, Istanbul, Turkey; Gazi University Hospital, Ankara, Turkey

**Keywords:** Lung adenocarcinoma, EML4-ALK, IGFR1, TTF1, Napsin A

## Abstract

***Objective:*** Patients with lung adenocarcinoma who harbor ALK gene rearrangements can demonstrate significant clinical benefit with ALK tyrosine kinase inhibitors. Insulin-like growth factor receptor 1 (IGFR1) is a cellular membrane receptor that is overexpressed in many tumors. It plays an important role in cancer progression and is associated with increased postoperative recurrence and poorer disease-free survival. The aim of this study was to determine the EML4-ALK mutation and IGFR1 expression in lung adenocarcinoma and analyze their prognostic value.

***Material and Method:*** In this study, we analyzed the EML4-ALK mutation using the FISH and IHC techniques in 251 lung adenocarcinoma (203 primary resections, 48 metastasectomies) cases. Correlative analyses were performed between the EML4-ALK mutation, the IGFR1, TTF1, and NapsinA expression, and the clinicopathologic factors in lung adenocarcinomas.

***Results:*** The EML4-ALK mutation was observed in 3.8% of the cases and it was associated with the solid pattern, signet ring cell morphology, and larger tumor size. IGFR1 expression was identified in 49% of the cases and most of the ALK-mutated cases were also expressing the IGFR1 protein (66%). IGFR1 expression frequency was increased in metastasectomy specimens.

***Conclusion:*** A solid signet-ring cell pattern or mucinous cribriform pattern was present at least focally in all ALK-positive tumors, consistently with the literature. In addition, IGFR1 expression levels showed an increase in the EML4-ALK-mutated cases in our series, but the clinical significance of this finding should be supported by larger series and survival analysis. Our findings show that IGFR1 expression may be useful as a poor prognostic marker in patients with lung adenocarcinoma.

## INTRODUCTION

Lung cancer is the most frequent cause of cancer deaths, accounting for approximately 1.6 million deaths per year worldwide (1). Primary lung cancer is mainly divided into two pathological types: small cell lung cancer (SCLC) and non-small cell lung cancer (NSCLC), of which NSCLC accounts for approximately 85% and adenocarcinoma is the most frequent histological subtype of NSCLC (2).

Many studies on lung cancer carcinogenesis have been conducted over the years. These studies are especially important for clinical treatment strategies and the development of targeted therapies. Intratumoral epidermal growth factor receptor (EGFR) mutation status has been especially found to be a strong predictive factor in lung adenocarcinomas (AC) for the efficacy of EGFR-tyrosine kinase inhibitors (TKI) (3). Translocation and inversion of the ‘Anaplastic lymphoma kinase‘ (ALK) gene with ‘Echinoderm microtubule-associated protein-like 4’ (EML4) has also been detected in a subset of non-small cell lung cancer (NSCLC) patients in 2007 (4). ALK mutations are present in approximately 3-6% of NSCLCs (5). Crizotinib is a first-generation TKI of ALK and it is the first drug with advanced ALK-positive NSCLC (6), and the patients can obtain effective results through treatment with inhibitors of ALK kinase (7). Although immunohistochemistry (IHC) is a nearly equivalent alternative to detect ALK mutation, fluorescent in-situ hybridization (FISH) technique still remains a reliable method for the diagnosis of ALK-rearranged fusions in NSCLC (8). Several studies have shown that certain patient characteristics such as younger age, never or light smoker, signet ring cell morphology, and adenocarcinoma subtype increase the probability of finding an ALK mutation (9,10).

Insulin-like growth factor receptor-1 (IGFR1) is a transmembrane protein implicated in promoting oncogenic transformation, growth, and survival of cancer cells (11) The overexpression of IGFR1 has been shown to correlate with postoperative recurrence and is associated with a poorer DFS in NSCLC patients (12). CT-707 is a mutant-selective inhibitor of ALK/focal adhesion kinase (FAK) and IGFR1 and it is designed to be a targeted therapeutic agent for NSCLC patients harboring ALK active and crizotinib-resistant mutations (13,14). However, to the best of our knowledge, there is no study in the literature showing whether there is a relationship between intratumoral IGFR1 expression and ALK mutation status in lung adenocarcinomas.

In this study, we investigated the histopathological features and clinical characteristics of patients with lung adenocarcinomas who harbored the EML4-ALK translocation in 251 lung resection (203 cases) and metastasectomy (48 cases) specimens. We used both the FISH and immunohistochemistry techniques for detection of ALK rearrangement. In addition, we analyzed the clinical and pathological role of IGFR1 expression and its association with the EML4-ALK mutation. Also, we examined the relationship between IGFR1, TTF-1 and Napsin-A expression, the ALK mutation, and the clinicopathological features (15,16).

## MATERIALS and METHODS

### Case Series

From 2006 to 2013, a total of 251 lung adenocarcinoma cases were included in our study. Two hundred three were pulmonary resection and 48 were lung cancer metastasectomy specimens. Clinical and pathological data were recorded using electronic medical files and pathology reports. The hematoxylin and eosin (H&E) stained slides were retrieved from the pathology archives and reviewed by two experienced pulmonary pathologists (PB, NA). The subtypes of adenocarcinoma were determined according to the International Association for the Study of Lung Cancer (IASLC) / American Thoracic Society (ATS) / European Respiratory Society (ERS) International Multidisciplinary Classification of Lung Adenocarcinoma (17). All samples were reclassified and updated according to the 2015 WHO classification and TNM staging (8th edition) for lung carcinomas (18). Tumor areas were selected and marked on hematoxylin & eosin stained sections. The slide was then overlaid on the original paraffin block to determine the corresponding area to be used. TMAs were constructed by using a 3 mm punch biopsy tool and, 2 representative cylindrical cores were taken from each tissue block and then arranged sequentially into a recipient paraffin block.

This study was supported by the ‘Gazi University Scientific Research Projects Unit’ in Turkey with the number of 01/2012-79. Ethics committee approval had been obtained on 23.05.2012 with decision number 213 at Gazi University, Turkey.

### EML4-ALK Analysis by FISH

### (Fluorescent In-Situ Hybridization) Technique

**Figure 1:**
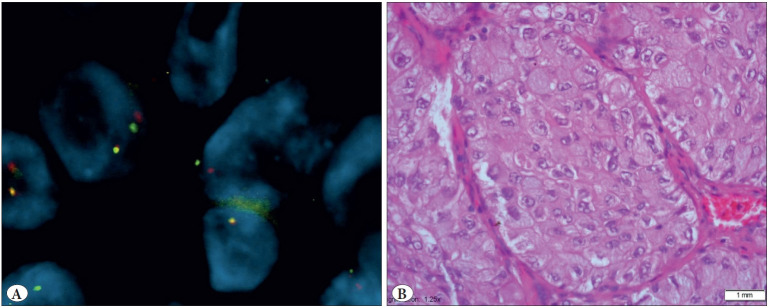
**A)** EML4-ALK gene rearrangement by FISH technique (FISH; x100). **B)** EML4-ALK positive tumor sample with solid pattern and signed ring cell morphology (H&E; x40).

To detect EML4-ALK rearrangement, the ‘Vysis Abbott Molecular ALK Break Apart FISH probe kit’ and ‘Paraffin Pretreatment Kit IV’ were used. The evaluation was performed with the BX51 Olympus fluorescence microscopy. The cell was regarded as positive when a nucleus had at least one set of broken apart signals, or had a single red signal (deleted green signal) in addition to fused and/or broken apart signals. The distance between two separate red and green signals was estimated using two times the biggest signal size. The samples were considered positive if more than 25 out of 50 tumor cells were positive, and negative if less than 5 tumor cells were positive. The sample with 5-25 positive tumor cells was considered equivocal, and was then evaluated by a second pathologist. If the average percent of the positive cells was 15% or more, the sample was considered positive. Otherwise, it was considered negative for ALK rearrangement (Figure 1).

### Immunohistochemistry

**Figure 2:**
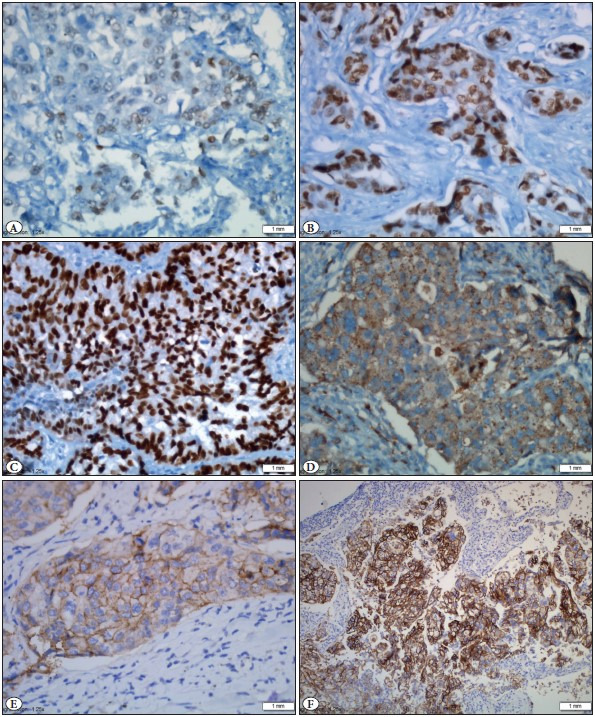
**A)** TTF1 nuclear positive staining; 1+ positive (IHC; x40). **B)** TTF1 nuclear positive staining; 2+ positive (IHC; x40). **C)** TTF1 nuclear positive staining; 3+ positive (IHC; x40). **D)** Napsin A cytoplasmic granular staining (IHC; x40). **E)** IGFR-1 membranous staining; 1+ positive (IHC; x40). **F)** IGFR-1 membranous staining; 2+ positive (IHC; x20).

The IHC staining of TMA sections was performed using the Ventana automated IHC staining system (Leica, Bond-Max). All the antibody labeling was detected using the 3,3´-diaminobenzidine (DAB) detection kit. Anti IGFR1 mouse monoclonal antibody was used against human IGFR1 (ab4065; Abcam, Cambridge, MA, USA), diluted 1:10 in PBS, and unstained slides were kept at room temperature for 45 minutes after boiling with EDTA for 20 minutes. IHC was considered positive when a distinct cell membrane staining was evident (Figure 2). Non-tumoral prostate tissue used for positive control. A cutoff value of 5% was used for the positivity rate and the positive cases were subclassified according to weak (1+) or strong (2+) staining rates.

TTF1 (Cell Marque, mouse monoclonal, clone 8G7G3/1; Ventana), and NapsinA (Cell Marque, NapsinA rabbit polyclonal; Ventana) were investigated on an automated immunostainer. The positive controls for TTF-1 labeling were non-tumoral lung parenchyma and alveolar macrophages were used for NapsinA. TTF1 expression was subclassified according to the staining intensity as high (3+), moderate (2+), and low (1+). NapsinA staining was considered positive when tumor cells had cytoplasmic granular staining at any intensity.

**Figure 3:**
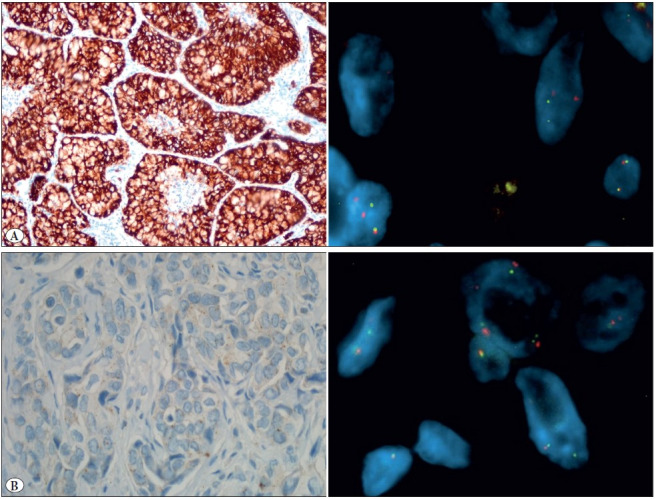
ALK IHC and ALK FISH. **A)** IHC positive FISH positive (IHC; x40) (FISH; x100). **B)** IHC negative FISH positive (IHC; x40) (FISH; x100).

IHC for ALK expression was performed wıth the D5F3 rabbit monoclonal antibody and ultrasensitive OptiView DAB IHC Detection Kit with amplification (Ventana anti-ALK (D5F3) IHC Assay). Strong granular cytoplasmic staining in tumor cells was defined as positive, and no staining was considered as negative (Figure 3).

**Statistical Analysis: **Student’s-t test was used for tumor size and age. Variables were analyzed, as appropriate, with the χ2 test, or the Mann-Whitney U test, to compare the differences in categorical variables. P value of less than 0.05 was considered statistically significant. The statistical analyses were carried out by using the SPSS for Windows 17.0 program (SPSS Inc., Chicago, IL).

## RESULTS

### Clinical Information

The baseline characteristics of resected lung adenocarcinoma cases are shown in Table 1. There were 203 patients in total; 150 (73.9 %) were women and 53 (26.1 %) were men. The female to male ratio was 2.8:1. The age ranged from 38 to 85 years. The mean age was 63 years, and the median age was 62 years. A hundred-sixty (78.8%) had a smoking history, and 43 cases (21.2 %) did not. According to the eighth edition of the TNM Classification of Lung Cancer (2015), the majority of the cases were stage I (49.8%), and 24 cases (11.8%) were stage IV.

**Table 1:** Characteristics of the ‘203 primary resection lung adenocarcinoma specimens’ and correlations between the patients harboring EML4-ALK mutation.

	**Total (%)**	**EML4-ALK Rearrangement**		**P**
		**Positive (%)**	**Negative (%)**	
**Age** (mean±SD, years)	63±8.84	62.11±8.89	62.4±12.2	0.500
**Gender** Male Female	150 (73.9) 53 (26.1)	3 (2) 2 (3.8)	147 (98) 51 (96.2)	0.608
**Size** mean±SD (cm)	3.7±2.5	5.54±3.34	3.61±2.02	**0.037**
**Pathological T-stage** T1A T1B T1C T2A T2B T3 T4	7 (3.4) 23 (11.3) 32 (15.8) 75 (37) 23 (11.3) 25 (12.3) 18 (8.9)			
**Pathological N-stage** Nx N0 N1 N2	37 (18.2) 108 (53.2) 37 (18.2) 21 (3.7)	1 (0.8) 2 (1.9) 11 (0.8) 1 (4.8)	36 (97.2) 106 (98.1) 36 (97.2) 20 (95.2)	0.611
**Visseral pleural invasion** Positive Negative	111 (54.7) 92 (45.3)	3 (2.8) 2 (2.2)	109 (97.2) 90 (97.8)	0.308
**Smoking history** Never Ever	43 (21.2) 160 (78.8)	3 (7) 2 (1.3)	40 (93) 158 (98.7)	0.064
**Stage** IA IB IIA IIB IIIA IIIB IV Unknown	47 (23.1) 52 (25.6) 23 (11.3) 14 (6.8) 22 (10.8) 3 (1.5) 24 (11.8) 18 (9.1)	Stage I 2 (2.1) Stage II, III, IV 3 (2.9)	Stage I 97 (97.9) Stage II, III, IV 101 (97.1)	0.489
**Dominant pattern** Acinar Papillary Lepidic Micropapillary Solid Fetal Mucinous Enteric Adenosquamous	118 (58.1) 29 (14.3) 6 (3.5) 4 (2) 30 (14.8) 1 (0.2) 12 (5.9) 1 (0.2) 2 (1)	Acinar 1 (2.4) Solid 4 (13.4)	Acinar 117 (97.6) Solid 26 (86.6)	**0.008**

### Clinical and Histological Correlations Between the Patients Harboring EML4-ALK Mutation

**Figure 4:**
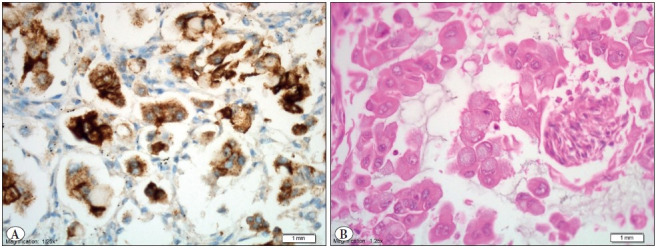
**A)** ALK positive tumor sample (IHC; x40) with **B)** signet ring cell morphology (H&E; x40).

We identified 9 patients who harbored the EML4-ALK fusion gene (3.9% (9/229) (Figure 1). Five (2.6%) of these positive cases, were lung resection specimens, and 4 (8.3%) were at metastasectomy specimens. Overall 13 cases were left out from the study as no fluorescent signal could be received. The mean tumor size of EML4-ALK positive cases was significantly larger than in EML4-ALK negative cases (mean±SD, 5.54±3.34 vs 3.61±2.02, 0.037) (Table 1). Of the FISH positive cases, the male-to-female ratio was 5:4, and the mean age was 58.2 (ranging from 48 to 81 years). A predominant solid/cribriform pattern was observed in 6 (66.6%) of the 9 cases, and a predominant acinar pattern in 3 cases (33.4%) (Table 2). A solid predominant pattern was also significantly found to be related with the EML-ALK mutation (0.008). More than half of the cases (5/9) showed signet ring cells (Figure 4). No significant differences were found in age, gender, smoking history, pathological N-stage, tumor location, and visceral pleural invasion with the EML-ALK translocation (>0.05) (Table 1).

**Table 2:** Detailed clinicopathological features of EML4-ALK-positive cases (Case 1 to 5 are from the primary resection, and Case 6 to 9 are from the metastasectomy specimens).

	**Case 1**	**Case 2**	**Case 3**	**Case 4**	**Case 5**	**Case 6**	**Case 7**	**Case 8**	**Case 9**
**Age**	81	66	48	58	59	59	64	53	77
**Gender**	Male	Male	Female	Male	Female	Female	Male	Male	Female
**Tumor size**	2,5 cm	9 cm	9 cm	5 cm	2.2 cm				
**Lymph nodes**	Nx	N2	N1	N0	N0				
**T stage**	IC	IV	IV	2B	IC	IV	IV	IV	IV
**Pleural invasion**	PL-0	PL-1	PL-1	PL-1	PL-0				
**Smoking**	No	Yes	No	Yes	No	No	Yes	Yes	Yes
**Dominant pattern**	Solid	Solid	Solid	Solid	Solid	Acinar	Acinar	Acinar	Solid
**TTF1**	2+	3+	2+	2+	2+	1+	3+	3+	2+
**Napsin A**	+	+	+	+	+	+	+	+	+
**IGFR**	2+	-	1+	1+	-	-	-	2+	1+
**ALK IHC**	3+	3+	3+	1+	3+	-	-	-	2+

**IHC:** Immunohistochemistry

Six of the 9 FISH-positive cases were also positive with IHC whereas 3 cases were negative with ALK IHC (Figure 3). Detailed clinical, histological and immunohistochemical findings of the EML4-ALK FISH positive cases are shown in Table 2.

### Correlation of the IGFR1 Expression and Clinicopathological Parameters

IGFR1 expression was identified in 123 (49%) of the 251 patients. There was no significant association between IGFR1 expression and the clinical parameters (Table 3).

**Table 3:** Characteristics of staining with TTF-1, Napsin-A and IGFR-1 in lung adenocarcinomas.

	**TTF-1 (%)**			**Napsin-A (%)**			**IGFR-1 (%)**		
	**Positive**	**Negative**	**p**	**Positive**	**Negative**	**p**	**Positive**	**Negative**	**p**
**Age** Mean±SD Median	61.75±8.86 62 (38-85)	62.78±8.87 64 (46-81)	0.81	61.66±9.1 62 (42-85)	62.5±8.33 63 (38-81)	0.211	61.7±8.9 60 (42-85)	62.2±8.84 63 (38-82)	0.888
**Sex** Female Male	47 (88.7) 114 (86)	6 (11.3) 36 (14)	**0.05**	45 (84.9) 86 (57.3)	8 (15.1) 64 (42.7)	**<0.001**	22 (41.5) 70 (46.7)	31 (58.5) 80 (53.3)	0.727
**Size** Mean±SD Median	3.4±1.90 3 (0.6-9)	4.6±2.31 4 (0.8-10)	**0.042**	3.3±1.85 2.85 (0.6-9)	4.3±2.2 3.6 (0.8-10)	**0.013**	3.93±2.16 3.2 (0.6-10)	3.41±1.93 3 (0.8-9)	0.165
**Node status** N0 N1 N2	82 (75.9) 30 (8.1) 18 (5.7%)	26 (24.4) 7 (18.9) 3 (14.3)	0.547	60 (55.6) 26 (70.3) 18 (85.7)	48 (44.4) 11 (29.7) 3 (14.3)	**0.018**	45 (42.9) 16 (43.2) 9 (42.9)	62 (57.1) 21 (56.8) 12 (57.1)	0.998
**Pleural inv** Positive Negative	86 (77.5) 75 (81.5)	25 (22.5) 17 (185)	0.493	65 (58.6) 66 (71.7)	46 (1.4) 26 (28.3)	0.051	99 (77.5) 88 (42.4)	7 (22.5) 9 (57.6)	0.445
**Pattern** Acinar+Papillary+Lepidic Solid+Mucinous Acinar Papillary Solid Mucinous Micropapillary Lepidic Fetal Enteric Adeno+squamous	125 (81.7) 28 (66.7) 93 (78.8) 26 (89.7) 21 (70) 7 (58.3) 4 (100) 6 (100) 1 (100) 1 (100) 2 (100)	28 (13.7) 14 (33.3) 25 (21.2) 3 (10.3) 9 (30) 5 (41.7) 0 (0) 0 (0) 0 (0) 0 (0) 0 (0)	**0.036**	105 (68.6) 19 (45.2) 74 (62.7) 26 (89.7) 15 (50) 4 (33.3) 4 (100) 5 (83.3) 1 (100) 1 (100) 1 (50)	48 (1.4) 23 (54.8) 44 (7.3) 3 (10.3) 15 (50) 8 (66.7) 0 (0) 1 (16.7) 0 (0) 0 (0) 1 (100)	**0.005**	68 (44.4) 22 (52.4) 56 (47.5) 7 (24.1) 13 (43.3) 9 (75) 0 (0) 5 (83.3) 0 (0) 0 (0) 1 (50)	85 (55.6) 20 (47.6) 62 (52.5) 22 (75.9) 17 (56.7) 3 (25) 4 (100) 6 (16.7) 1 (100) 1 (100) 1 (50)	0.361
**Stage** I IA IB II IIA IIB III IIIA IIIB IV	88 (84.2) 47 (89.4) 41 (78.8) 28 (66.7) 16 (69.6) 8 (57.4) 23 (76.7) 18 (81.8) 2 (66.7) 19 (79.2)	16 (15.8) 5 (10.6) 11 (21.2) 13 (33.3) 7 (30.4) 6 (42.6) 5 (23.4) 4 (18.2) 1 (33.3) 5 (20.8)	0.113	65 (64.4) 34 (72.3) 30 (52.7) 24 (57.1) 15 (65.2) 6 (42.9) 22 (73.3) 17 (77.3) 2 (66.7) 16 (6.7)	36 (35.6) 13 (27.7) 22 (47.3) 18 (47.9) 8 (34.8) 8 (57.1) 8 (26.7) 5 (227) 1 (33.3) 8 (33.3)	0.559	43 (42.6) 47 (31.7) 46 (48.1) 16 (38.1) 22 (26.1) 13 (57.1) 16 (53.1) 11 (50) 3 (33.3) 14 (58.3)	58 (7.4) 5 (68.3) 11 (51.9) 26 (61.9) 7 (73.9) 6 (42.9) 14 (46.7) 4 (50) 1 (66.7) 10 (41.7)	0.307
**Smoking** Ever Never	124 (78.7) 35 (81.4)	34 (21.3) 8 (186)	0.704	98 (61.3) 33 (76.7)	62 (38.7) 10 (23.3)	0.059	76 (47.5) 16 (37.2)	84 (52.5) 27 (62.8)	0.301

### Relationship Between IGFR1 Expression and Metastasis Risk

According to our results, the IGFR1 expression rate was significantly higher in metastasectomy specimens (45.2% vs 81.2%) (0.02). At the same time, IGFR1 staining intensity was stronger (2+) than in the primary resections (7.9% vs 35.4%) (Table 4). These results explain the effect of IGFR1 expression on the prognosis. Besides these results, IGFR1 expression was not associated with any clinicopathological characteristics in primary tumors as well as metastasectomies (Table 3).

**Table 4:** IGFR1 staining rates between metastasectomy and resection cases.

	**IGFR1 (-)**	**IGFR1 (1+)**	**IGFR1 (2+)**	**Total (+)**	**p**
Resection	111 (54.7)	76 (37.4)	16 (7.9)	92 (45.2)	0.02
Metastasectomy	17 (18.8)	22 (45.8)	9 (35.4)	31 (81.2)	

### Relationship Between IGFR1 Expression and EML4-ALK Mutation

Most of the ALK mutated cases were also expressing the IGFR1 protein (66%). However, this result was not statistically significant. This may be due to the small number of EML4-ALK-positive cases (>0.05).

### Correlation Between TTF1 and Napsin A Expression and Clinicopathological Parameters

### TTF-1 Expression and Clinicopathological Parameters

TTF1 expression was seen in 202 (80.5%) (79.3% resection, 85.4% metastasectomy) cases, and 49 (19.5%) cases were negative for TTF1. TTF1 expression had a significant correlation with the female sex and it was more frequently seen in the acinar, papillary, and lepidic patterns (81.7%) rather than solid and mucinous patterns (66.7 %) (0.036). Likewise, TTF-1 expression was more frequent in smaller tumors than the larger ones (0.042). Furthermore, there was no significant association of TTF1 expression with age, lymph node involvement, visceral pleural invasion, stage, and smoking status, in both primary cases and metastasectomies (>0.05) (Table 3). We could not find a relation between expression intensity and the histological patterns, but most acinar and papillary patterns and all lepidic, micropapillary, fetal, and enteric patterns had intense (3+) expression, while others had a lower expression intensity (1+, 2+) expression with TTF1 (No data shown).

### Napsin A Expression and Clinicopathological Parameters

Napsin A expression was seen in 74% (74.9% resection, 66.7% metastasectomy) of our cases. Napsin A expression was associated with the female sex (<0.001), lymph node metastasis (0.018), and the tumor size (0.013). The expression rate of Napsin A significantly decreased with tumor size (Table 3). Similar to the TTF1 expression, Napsin A expression was also more frequent in well-differentiated patterns (acinar, papillary, and lepidic) rather than solid and mucinous patterns in both primary resection (68.6%) (0.048) and metastasectomy specimens (92.3%) (0.002). Napsin A expression was not correlated with age or smoking.

## DISCUSSION

Personalized targeted therapy has emerged as a promising strategy in lung cancer treatment. Several oncogenic drivers have been reported in this context. ALK rearrangement is responsible for about 3-6% of all non-small cell lung cancers. Although it is seen in only a small percentage of lung cancers, it is an important predictor of response to anti-ALK targeted therapies (19). Selection of patients based on their ALK status is therefore vital, on account of the high response rates with the ALK targeted agents. In our study, we used the ALK FISH assay, which is accepted as the gold standard in the assessment of ALK gene rearrangement (20). We found the overall frequency of ALK mutation to be 3.8%, which was almost consistent with previous reports. The ALK mutation rate decreases with increasing patient age in most studies (21,22). In our study, we could not find a statistical correlation with age, but the median age of our positive cases was younger than the negative ones as in the literature (59 vs. 63). Shaw et al. (23) noted that the rate of EML4-ALK fusion gene mutation is higher in men than in women (22.9% vs. 8.6%). Similarly, the mutation risk is higher in men than in women, but we did not find a statistical correlation. Some studies have shown that ALK-positive adenocarcinomas were more common in patients at advanced T stages (22,24). Consistently, our ALK positive cases had a larger tumor size (median 5 cm vs. 3 cm) than the negative ones (0.037).

Our results further indicate that the EML4-ALK translo-cation occurs mostly in solid predominant and signet ring cell morphology tumors (0.008), and this finding is consistent with the results of other published studies. Rodig SJ. et al. demonstrated the pattern relationship with EML4-ALK translocation in lung adenocarcinomas (25). Similarly, the EML4-ALK translocation was observed more frequently in cases with solid or acinar growth patterns, cribriform structure, mucous cells (signet-ring cells, or goblet cells), and abundant extracellular mucus, and also in those lacking lepidic growth and significant nuclear pleomorphism in a different study (26).

We could not find any statistically significant correlation between TTF1, NapsinA, or IGFR1 expression and the EML4-ALK translocation.

The present study revealed a significant finding regarding IGFR1 expression. IGFR1 expression was correlated with increased metastasis risk. These results indicate that IGFR1 expression can be used to indicate a poor prognosis. Nakagawa et al. have shown that high IGFR1 expression is associated with increased postoperative recurrence and poorer disease-free survival (12). Many studies share the correlation of IGFR1 expression with cell survival, growth, proliferation and angiogenesis, as well as blocking of apoptosis, and it is also linked with many cancers in different organs (27,28). In a recent study blocking ALK and IGFR1 receptors together with the CT-707 drug, which is one of the FAK (focal adhesion kinase) inhibitors, significantly inhibited tumor growth without obvious side effects (13,29). Overexpression of IGF1R and FAK are closely associated with metastatic breast tumors (30). Our results indicate increased levels of IGFR1 expression in metastasectomy specimens. However, we could not find a relationship with the other prognostic parameters, maybe due to the low number of cases in our study.

Few studies have implicated a correlation between TTF1 positivity by IHC and EGFR, KRAS mutation status (31). However, the role of TTF1 in lung cancer pathogenesis and its relationship with the ALK translocation status is unclear. TTF1 and NapsinA are mainly used as diagnostic markers of lung adenocarcinomas in daily practice. In our cohort, 80% of the cases were positive with TTF1 and/or NapsinA. In addition, 33 cases (13%) were negative with both TTF1 and NapsinA. These cases were diagnosed as pulmonary adenocarcinoma based on the morphological features and the clinical ruling out of other possible primaries with IHC. Our results showed that TTF1 and NapsinA expression were associated with the female sex and smaller tumor sizes. Napsin A expression was also related to a good prognosis because of its relationship with the N0 cases. These results are consistent with the literature (32).

In our study, we investigated the correlation of TTF1 and NapsinA expression by IHC with EML4-ALK mutations and also with the clinicopathological characteristics. According to our results, all EML4-ALK mutated cases were positive with TTF1 and NapsinA (100%). This result is consistent with the other similar study in the literature (33). Inamura et al. explained the TTF1 positivity of EML4-ALK lung cancers with the ‘terminal respirator unit (TRU) histogenesis. TRU-type lung cancers with a TTF1 positive cell lineage often occur in non or light smokers, which frequently harbor EGFR mutations (61%) and have less-frequent TP53 mutations (36%) compared to non-TRU-types (57%) (33,34).

## CONFLICT of INTEREST

The authors declare no confict of interest.

## FUNDING

This study was funded by the ‘Gazi University Scientific research projects unit’ in Turkey with the number of 01/2012-79.
